# Colonic leiomyosarcoma with lung metastasis or lung cancer: A case report

**DOI:** 10.1002/ccr3.9011

**Published:** 2024-11-24

**Authors:** Tongkun Song, Kai Xu, JiaDi Xing, Maoxing Liu, Fei Tan, Xiangqian Su

**Affiliations:** ^1^ Key Laboratory of Carcinogenesis and Translational Research (Ministry of Education), Department of Gastrointestinal Surgery IV Peking University Cancer Hospital and Institute Beijing China

**Keywords:** case report, colonic leiomyosarcoma, desmin, lung adenocarcinoma, lung metastasis

## Abstract

Colonic leiomyosarcoma is a tumor with a very low incidence and a high metastasis rate, mainly lung metastasis. This report provides insights into the future treatment. Thoracic puncture is necessary for patients with pulmonary nodules.

## 1 INTRODUCTION

Colonic leiomyosarcoma (CLMS) is a malignant tumor originating from muscularis mucosa or propria. CLMS has been clearly classified since 1998 when Hirota et al. discovered CD117 (KIT).^1^ and separately grouped as gastrointestinal stromal tumors (GISTs). Studies on CLMS are limited and most report individual cases, making it difficult to determine the incidence of CLMS. Moreover, the etiology of CLMS is unclear; however, few studies have revealed that most cases are related to immunosuppression.^2^, ^3^

Patients generally have no specific symptoms. However, the rate of metastasis, particularly lung and liver metastasis, is high.^4^ Diagnosis based on imaging is also very difficult due to the nonspecificity of imaging features.^3^, ^4^ Unfortunately, histological biopsy has a high error rate due to the origin of the tumor in the muscularis, and the preoperative diagnosis rate is only 29%.^5^ The tumor is mainly composed of heterotypic spindle cells. Immunohistochemical findings are positive for desmin, h‐caldesmon, and smooth muscle actin (SMA) and negative for CD117, CD34, and discovered on GIST‐1 (DOG1).^2^, ^6^, ^7^

Surgical excision is the preferred treatment option in the clear absence of distant metastasis. Adjuvant chemotherapy and radiotherapy are not routinely administered because of tumor tolerance.^6^ CLMS is a highly aggressive tumor with a poor prognosis.^2^, ^6^

## 2 CASE PRESENTATION

**FIGURE 1:**
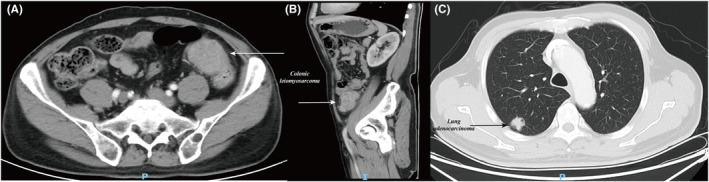
Contrast‐enhanced computed tomography of the chest, abdomen, and pelvis.

**FIGURE 2:**
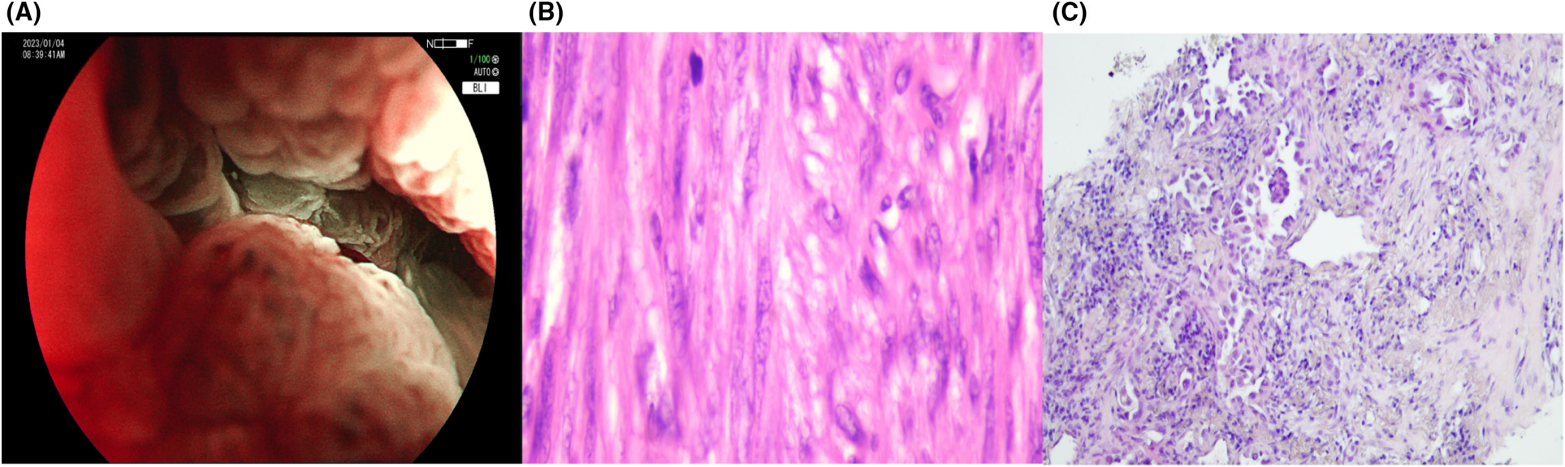
A, Colonoscopy showed circumferential constriction of the descending colon, and could not pass through this narrow bowel cavity. B, Colonic lesion biopsy (hematoxylin–eosin staining). C, Pulmonary lesion biopsy (hematoxylin–eosin staining).

A 63‐year‐old male patient with no past history had dull pain in the left lower abdomen for >1 year and abdominal distension and difficulty in defecation for 3 months. There was no history of weight loss or anorexia, and the patient did not have any chronic diseases or comorbidities. Additionally, the patient's personal, psychosocial, and family history were unremarkable. Vital signs and general physical examination were normal. The abdominal inspection, auscultation, and percussion yielded no abnormal findings. Abdominal palpation revealed a firm fixed globular 6*5*4 cm mass with mild tenderness in the left lower abdomen. Digital rectal examination was normal. Hematological examination revealed a carcinoembryonic antigen level of 6.18 μg/L. Chest, abdomen, and pelvic cavity contrast‐enhanced computed tomography (CT) scan revealed a thickened intestinal wall of the descending colon with soft tissue mass formation, which was considered malignant (Figure 1A,B). The subpleural nodules in the posterior segment of the upper lobe of the right lung indicated a high possibility of lung cancer (Figure 1C). Positron emission tomography/CT revealed hypermetabolism at same sites, which were considered malignant, with no other metastases. Colonoscopy combined with pathological biopsy revealed some heterotypic spindle cells, desmin (+), SMA (+), CD34 (−), CD117 (−), DOG1 (−), and S100 (−), consistent with leiomyosarcoma (Figure 2A,B).

## 3 METHODS

**FIGURE 3:**
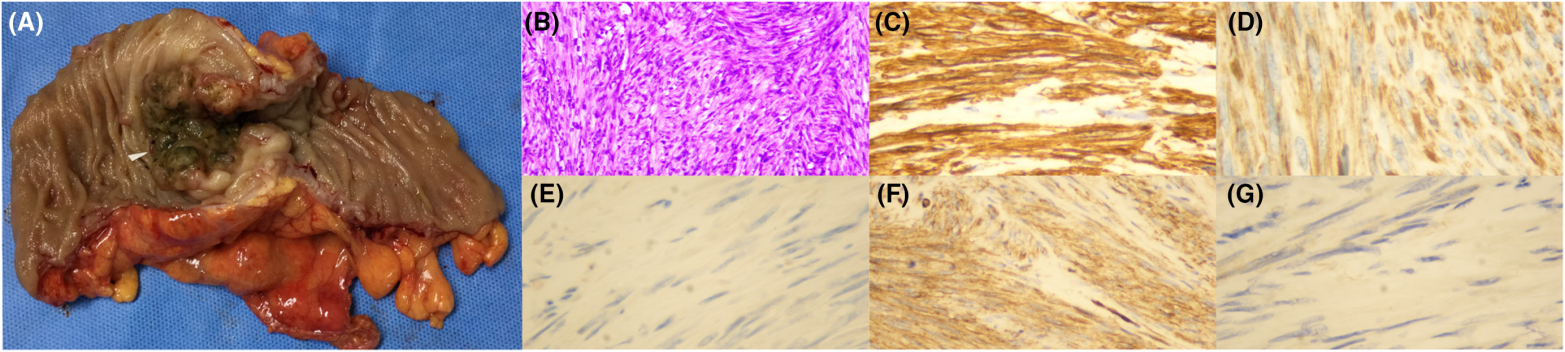
A, Postoperative specimen of the descending colon. B, Pathological section of the descending colon specimen (hematoxylin–eosin staining). C, Immunohistochemistry, SMA (+). D, Immunohistochemistry, desmin (+). E, Immunohistochemistry, CD117 (−). F, Immunohistochemistry, CD34 (−). G, Immunohistochemistry, DOG1 (−).

The most common site of CLMS metastasis is the lung. The relationship between the two lesions (primary with metastasis or double primary) was considered to affect the treatment choice after a multidisciplinary discussion. CT‐guided chest puncture was perfected and pathological findings revealed thyroid transcription factor 1 (TTF1) (+), CD56 (−), P40 (−), and adenocarcinoma (Figure 2C). The colonic lesion was planned to be first treated followed by the pulmonary lesion, considering that the patient had colonic incomplete obstruction. Laparoscopic‐assisted left hemicolectomy was performed (Figure 3A). The results of postoperative pathology were consistent with those of preoperative biopsy (Figure 3B–G). No metastasis was observed in the lymph nodes (0/19). The patient recovered successfully and was discharged on the fifth day postoperatively. The lung surgery proceeded as planned and is currently in a satisfactory recovery state.

## 4 CONCLUSION AND RESULTS

The patient has been regularly followed up for 1 year post‐surgery, and there have been no findings of recurrence or metastasis. Thoracic puncture is necessary for patients with CLMS with pulmonary nodules. The distinction between primary lung tumors and lung metastases can directly influence the subsequent tumor treatment approach.

## 5 DISCUSSION

CLMS has a very low prevalence, with a lack of treatment strategies and a very high incidence of metastasis, especially in the lungs. Clinical manifestations, imaging findings, and pathological findings are commonly utilized for distinguishing primary lung cancer from secondary lung metastases. The typical symptoms of primary lung cancer include cough, sputum production, shortness of breath, and chest pain. In contrast, secondary lung metastases are often accompanied by the symptoms of the primary tumor. On chest radiography or CT examination, primary lung cancer typically presents as a lung mass or shadow, while secondary lung metastatic cancer manifests as multiple pulmonary metastatic nodules. These nodules typically appear in various sizes. However, distinguishing solitary metastatic pulmonary nodules from primary pulmonary nodules in the early stage is a highly challenging task. Lung biopsy is considered the gold standard for accurately diagnosing the type and origin of lung cancer.^8^, ^9^, ^10^ AI‐assisted colonoscopy (AIC) has significantly enhanced the detection rate of colonic masses. It is anticipated that in the future, more advanced AIC will play a crucial role in assisting with the prediction and diagnosis of pathological types, further improving patient care and outcomes.^11^ Alvite et al. reported a case of lung metastasis as the first CLMS manifestation.^12^ Zhou et al. identified multiple lung metastases and colon lesions using PET/CT. Subsequently, they confirmed the presence of colon leiomyosarcoma with lung metastasis through a lung puncture biopsy and colonoscopy. The distinguishing factor in this case is the presence of multiple lesions in the lungs, which supports the diagnosis of metastasis.^13^ Although there is no single guideline for the management of colorectal leiomyosarcoma, the experience in the treatment of soft tissue sarcomas is referenced. At present, radical surgery is the primary treatment option for nonmetastatic CLMS.^6^, ^14^, ^15^, ^16^ Rowe et al. performed local resection in patients who refused radical surgery. However, the tumor recurred 7 months postoperatively, and the patients underwent another local resection and a course of external beam radiotherapy. Local excision plus radiotherapy may be an alternative to radical surgery.^17^ Anthracyclines are recommended as the first‐line treatment for patients with high‐burden and high‐grade tumors.^18^ Bennassi et al. preoperatively administered radiotherapy to control and shrink the lesions. The scope of surgical resection was significantly reduced.^19^ In general, leiomyosarcoma is less sensitive to radiotherapy and chemotherapy. Immunotherapy and targeted therapy have been attempted for CLMS. Vascular endothelial growth factor (VEGF) and c‐Met play a crucial role in promoting tumor angiogenesis, proliferation, and invasion. The use of multikinase inhibitors and monoclonal antibodies targeting these two molecules has shown promising results in the treatment of cancer.^20^ Hong et al. detected breast cancer (BRCA) mutation and rearrangement in patients with CLMS using next‐generation sequencing. Patients gained clinical benefits for 1 year following the administration of targeted therapy olaparib.^21^ Tay et al. observed pathological complete responses in patients with leiomyosarcoma with a high level of microsatellite instability, high tumor mutational burden (10 mutations/Mb), and significant programmed death ligand‐1 protein expression treated with immunotherapy.^22^ Chen et al. detected significant tumor regression with a combination of local radiation therapy and the anti‐PD‐1 antibody nivolumab.^23^ The case reported herein is that of a patient with a primary lung mass. As no significant lymph node metastasis was observed, a thoracoscopic lobectomy was performed directly 1 month following intestinal surgery. The prognosis after lung surgery is predicted to be better than that of lung metastasis due to the patient's active treatment and chest CT puncture. The patients hold the belief that adhering strictly to the doctor's advice regarding treatment will facilitate disease recovery. This case report provides more insights into subsequent CLMS diagnosis and treatment.

## AUTHOR CONTRIBUTIONS

**Tongkun Song:** Conceptualization; project administration; writing – original draft; writing – review and editing. **Kai Xu:** Conceptualization; supervision; validation. **JiaDi Xing:** Project administration; validation. **Maoxing Liu:** Project administration; validation; visualization. **Fei Tan:** Project administration; validation; visualization. **Xiangqian Su:** Conceptualization; project administration; supervision; validation.

## FUNDING INFORMATION

This work was supported by the National Natural Science Foundation of China (no. 82171720 and 81872022), Beijing Hospitals Authority Clinical Medicine Development of Special Funding Support (no. ZYLX202116), and Clinical Research Fund for Distinguished Young Scholars of Beijing Cancer Hospital (no. QNJJ2022019).

## CONFLICT OF INTEREST STATEMENT

The authors have no conflict of interest to declare.

## ETHICS APPROVAL STATEMENT

Our institution does not require ethical approval for reporting individual cases or case series.

## CONSENT

Written informed consent was obtained from the patient to publish this report in accordance with the journal's patient consent policy.

## Data Availability

The datasets used and/or analyzed during the current study are available from the corresponding author on reasonable request.
